# Space and Time Resolved Detection of Platelet Activation and von Willebrand Factor Conformational Changes in Deep Suspensions

**DOI:** 10.1155/2017/8318906

**Published:** 2017-11-06

**Authors:** Jacopo Biasetti, Kaushik Sampath, Angel Cortez, Alaleh Azhir, Assaf A. Gilad, Thomas S. Kickler, Tobias Obser, Zaverio M. Ruggeri, Joseph Katz

**Affiliations:** ^1^Department of Mechanical Engineering, Johns Hopkins University, 3200 N. Charles Street, Baltimore, MD 21218, USA; ^2^Department of Radiology and Radiological Science, Johns Hopkins School of Medicine, 1550 Orleans Street, Baltimore, MD 21287, USA; ^3^Department of Biomedical Engineering, Johns Hopkins University, 3200 N. Charles Street, Baltimore, MD 21218, USA; ^4^Department of Pathology, Johns Hopkins School of Medicine, 1800 Orleans Street, Baltimore, MD 21287, USA; ^5^Department for Pediatric Hematology and Oncology, University Medical Center Hamburg-Eppendorf, 20246 Martinistraße 52, 20251 Hamburg, Germany; ^6^MERU-Roon Research Center for Vascular Biology, Department of Molecular Medicine, The Scripps Research Institute, 10550 N. Torrey Pines Rd, La Jolla, CA 92037, USA

## Abstract

Tracking cells and proteins' phenotypic changes in deep suspensions is critical for the direct imaging of blood-related phenomena in* in vitro* replica of cardiovascular systems and blood-handling devices. This paper introduces fluorescence imaging techniques for space and time resolved detection of platelet activation, von Willebrand factor (VWF) conformational changes, and VWF-platelet interaction in deep suspensions. Labeled VWF, platelets, and VWF-platelet strands are suspended in deep cuvettes, illuminated, and imaged with a high-sensitivity EM-CCD camera, allowing detection using an exposure time of 1 ms. In-house postprocessing algorithms identify and track the moving signals. Recombinant VWF-eGFP (rVWF-eGFP) and VWF labeled with an FITC-conjugated polyclonal antibody are employed. Anti-P-Selectin FITC-conjugated antibodies and the calcium-sensitive probe Indo-1 are used to detect activated platelets. A positive correlation between the mean number of platelets detected per image and the percentage of activated platelets determined through flow cytometry is obtained, validating the technique. An increase in the number of rVWF-eGFP signals upon exposure to shear stress demonstrates the technique's ability to detect breakup of self-aggregates. VWF globular and unfolded conformations and self-aggregation are also observed. The ability to track the size and shape of VWF-platelet strands in space and time provides means to detect pro- and antithrombotic processes.

## 1. Introduction

Blood cells and proteins are structurally and functionally affected by abnormal flow conditions caused by blood-contacting devices [1] and cardiovascular pathologies, such as aortic stenosis [2], which frequently lead to abnormal clotting and bleeding [3]. Quantification of these changes, both* in vitro *and* in vivo*, is commonly based on bulk measurements, such as flow cytometry [4, 5]. While providing vital information on the extent of the blood response to abnormal conditions, these measurements cannot pinpoint the locations and temporal evolution of these processes. There is a pressing need for imaging techniques capable of capturing blood phenomena in deep suspensions. For example, inside transparent replica of mechanical circulatory support devices, like left ventricular assist devices and total artificial hearts, abnormal conditions are also present deep inside the fluid volumes under investigation, for example, in the bearing region between the inlet guide vane and the rotor, and in the central jet of a prosthetic valve. Therefore, this study introduces space and time resolved imaging procedures for in situ quantification of key biological phenomena involved in the blood response to abnormal hemodynamics in deep volumetric suspensions. Specifically, we focus on platelet activation, von Willebrand factor (VWF) conformational changes, and VWF-platelet interaction. The techniques are applicable to arbitrary geometries, to virtually all cell and protein types, and to extremely low signal intensities associated with high flow rates through clinically relevant systems, such as blood-contacting devices and cardiovascular system replica. Hence, they provide a flexible tool for identifying local flow mechanisms contributing to blood damage.

Activated platelets express the cell adhesion molecule P-Selectin (CD62P) [6] on their external surface, and its detection is routinely used to quantify platelet activation levels via flow cytometry using dye-conjugated anti-P-Selectin antibodies [7]. In this work, we employ a Fluorescein Isothiocyanate- (FITC-) conjugated anti-human P-Selectin antibody to detect activation of free moving platelets in deep suspensions. Moreover, the progression of activation is characterized by an increase in the concentration of intracellular calcium ions ([Ca^2+^]_i_) [8, 9].* In vitro *measurements of platelet activation have been performed using several Ca^2+^-sensitive probes via flow cytometry [10] and fluorometry [11], as well as using epifluorescence microscopy [8, 9]. Fluorescence measurements of platelets' Ca^2+^ fluxes in a cone-and-plate viscometer using the Ca^2+^-sensitive probe Indo-1 have also been performed [12]. It has been shown [13] that the increase in platelet's [Ca^2+^]_i_ is detected approximately 200 ms after thrombin stimulation and lasts from several seconds to minutes [14]. In this paper, Indo-1 [12, 15] is used to detect the increase in the number of platelets with detectable levels of [Ca^2+^]_i_ after stimulation, indicating ongoing activation.

The multimeric glycoprotein VWF plays a crucial role in platelet adhesion and aggregation under shear [16–19]. VWF transitions from a globular to an unfolded structural conformation upon exposure to mechanical stresses exceeding a threshold value [20, 21]. In its unfolded configuration, VWF exposes several functional domains allowing for platelet binding and for cleavage by the ADAMTS13 metalloprotease [22]. VWF functionality is size-dependent and cleavage of the VWF multimers reduces their adhesive potential [16]. Abnormally high shear stresses can result in pathological levels of cleavage, in turn leading to bleeding [2, 23, 24].* In vitro* visualizations of VWF dynamics have employed microfluidic devices and focused largely on wall-bound VWF [25–27]. For example, anchored VWF strings have been visualized after perfusion of fluorescently labeled platelets [26] or using an FITC-conjugated polyclonal anti-VWF antibody (VWF : IgG : FITC) [25]. Free flowing fluorescently labeled VWF has been visualized using wide-field, single-molecule fluorescence [28] and standard fluorescence microscopy [29]. VWF conformational changes in response to shear have been quantified using small-angle neutron scattering [30] and static and dynamic light scattering [31]. These observations have demonstrated the complex interactions between VWF and the flow but have been confined to shallow samples, 30–200 *μ*m in depth. Another approach, which provides full control of the number of fluorophores labeling the VWF multimers, involves a recombinant VWF with one eGFP attached to each monomer (rVWF-eGFP) [32]. To date, wall-bound rVWF-eGFP has been visualized only under static conditions. In this work, the number of rVWF-eGFP multimers is used to characterize the breakup of VWF aggregates after brief exposures to high shear stresses, while their shape is used to determine their conformational configuration in deep suspensions.

In addition, human VWF labeled with an IgG : FITC is used for visualizing suspended moving platelet-decorated VWF aggregates. The ability to identify and track such aggregates could potentially be used for quantifying aggregate breakup and prothrombotic processes induced by chemical agonists and mechanical stresses.

## 2. Methods

### 2.1. Optical Setup

**Figure 1:**
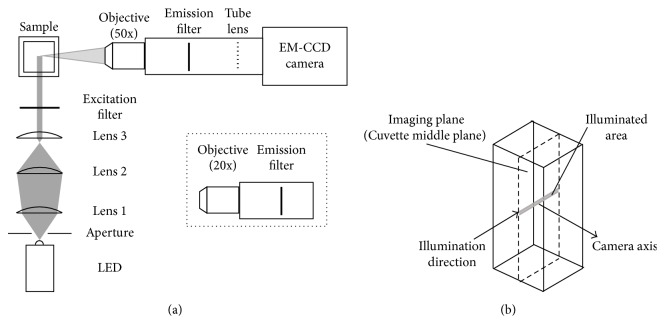
(a) Optical setup employed for the imaging of platelet activation, rVWF-eGFP, and platelet-decorated VWF : IgG : FITC strands. (b) Imaging area location inside the cuvette.

The setup shown in Figure 1(a) is used for acquiring 16-bit images with a high-speed, high-sensitivity, 1024 × 1024 pixels with 13 *μ*m pixel size, EM-CCD camera (iXon Ultra 888, Andor, Belfast, Northern Ireland) operating at −60°C with an EM gain of 750. A 50x Plan Apo SL, long-range, infinity corrected objective (Mitutoyo America) is used for rVWF-eGFP and VWF-platelet imaging, with a field of view (FOV) of 266 × 266 *μ*m^2^. A 20x DIN Achromatic objective (Edmund Optics, NJ) is used for platelet visualization, with a FOV of 665 × 665 *μ*m^2^. The bandpass excitation and emission filters specified in Table 1 are used for selecting the transmitted wavelengths. Most of the images are recorded at 16 frames per second (fps) with a 60 ms exposure time. This exposure is sufficient for detecting signals, yet it is short enough to prevent formation of motion-induced streaks in the absence of externally forced flow. The absence of motion-induced artifacts is confirmed by comparing the size of cells and proteins imaged at 60 ms with the ones observed under static conditions. However, to demonstrate the ability to detect VWF and platelets using very short exposure times, which would be required for visualizations in high-speed flows, such as stenotic jets, images are also recorded with a 1 ms exposure time, at 23 fps (this frame rate is needed to maintain the full field of view). The samples, contained in disposable 1 × 1 × 4.3 cm^3^ UV transparent cuvettes (SpectrEcology, FL), are illuminated at 90° by collimated LED beams (Thorlabs, Inc.) of the wavelengths indicated in Table 1. An aperture to limit the beam angle and lenses to collimate it are employed. The images are acquired at a fixed plane located near the center of the sample, 5 mm from the cuvette front and back walls.

### 2.2. Postprocessing Algorithms

**Figure 2:**
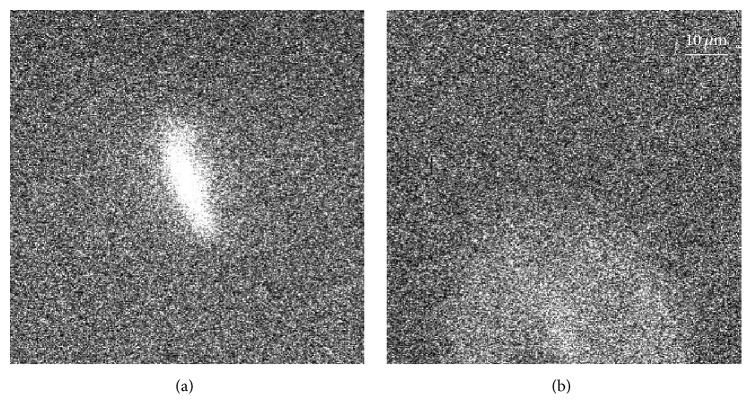
Contrast adjusted sample signals from the same moving rVWF-eGFP at different times, where the protein is (a) only partially in focus and (b) illuminated but slightly out of focus.

Because imaging is performed in deep suspensions, all the analyzed cases contain (i) images where only part of the protein/cell is in focus, for example, when the object is larger than the depth of focus (e.g., Figure 2(a)), and (ii) out-of-focus signals located within the illuminated field, close enough to the imaging plane to appear as broadened distinct signals (Figure 2(b)). Both events require particular care during postprocessing, which is performed using in-house Matlab codes, as summarized below. It is important to emphasize that, because of inherent resolution limitations, all the fluorescent signals are larger than the corresponding size of the cell/protein.

#### 2.2.1. rVWF-eGFP

The out-of-focus signals introduce background nonuniformities varying in space and time. The signal detection and tracking strategy relies on the suspension being dilute: the number of signals in a single image (1–5) and the rate of signals crossing a certain pixel, at most 1 per 1.5 s, are low. The postprocessing consists of two steps: (i)* image enhancement,* where as much background noise as possible is removed and the signal-to-noise ratio (SNR) is increased, and (ii)* identification and tracking* of the multimers across images.

**Figure 3:**
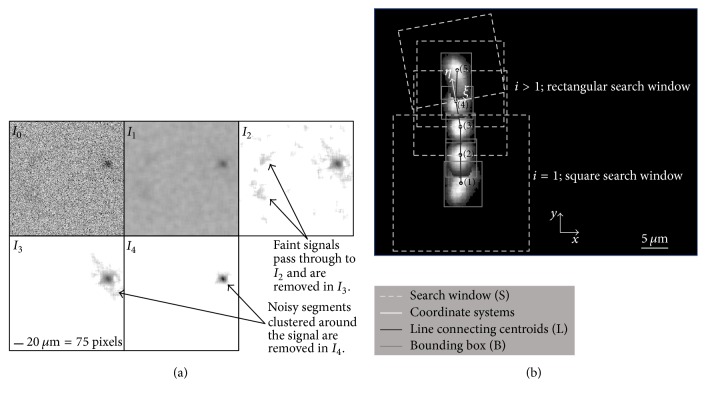
(a) rVWF-eGFP image enhancement procedure. *I*_0_, inverted raw image after contrast adjustment; *I*_1_, median filtered image; *I*_2_, temporal background subtracted image; *I*_3_, spatial filtered image; *I*_4_, final image after particle specific enhancement (PSE). (b) Superposition of multiple frames of an rVWF-eGFP enhanced signal demonstrating the signal identification and tracking algorithm.

*(i) Image Enhancement.* Figure 3(a) illustrates the four-step image enhancement procedure, with the gray scale inverted for clearer visualization.

These four steps are detailed as follows.

(1) Each image is contrast adjusted to obtain *I*_0_, followed by median filtering using a 20 × 20 pixels window, which results in *I*_1_. This step enhances the SNR and removes the salt-and-pepper noise. The median filter size is manually adjusted to the minimum value required for smoothing out noise without smearing the signals [33]. Factors affecting the filter size included the EM-CCD camera settings (EM gain, readout speed, etc.), magnification, and signal intensity. This approach is adopted throughout this section unless otherwise noted.

(2) Noise is further removed by a weighted background subtraction based on the running average *μ*_*l*_^*h*^ and standard deviation *σ*_*l*_^*h*^ of each pixel intensity over 2*l* + 1 images, with *l* = 15 for the current datasets. To prevent the inclusion of the fluorescent signal in the background calculation, the average and standard deviation are determined using pixel values shifted in time by *h* frames, that is, the maximum signal duration in the FOV, which is 20 for the present analysis. The signal is then enhanced according to

(1) $$\begin{array}{c} I_{2}\left(\;x,y,i\;\right)=I_{1}\left(\;x,y,i\;\right)-\left(\;k_{1}\mu_{l}^{h}+k_{2}\sigma_{l}^{h}\;\right), \end{array}$$

where *x*, *y*, and *i* represent the pixel coordinates and the image number. The values of *k*_1_ and *k*_2_ are constant for each dataset and are based on the background intensity but can vary across sets to account for differences in SNR. As *I*_2_ in Figure 3(a) shows, this step removes most of the background noise, although some patches still remain. This approach is not effective in cases involving large spatial and temporal variations in image intensity, for example, at high concentrations with multiple out-of-focus signals. In such situations, a 90 × 90 pixels spatially median filtered image is used as background and subtracted from each pixel value. The size of the filter is manually adjusted until the value required to remove the intensity variations while keeping the background pattern as intact as possible is found.

(3) Most of the remaining noise is removed from *I*_3_ by offsetting to zero pixels whose intensity falls below a threshold level determined based on a linear combination of the spatial mean and standard deviation of the instantaneous intensity distribution. The associated constants are adjusted for each dataset.

(4) Following segmentation, which provides the smallest rectangle containing the signal, a particle specific enhancement (PSE) algorithm is used to individually enhance each particle by gamma correction [34]. In this process, the particle signal is biased toward high intensities values, with the value of the parameter *γ* based on the intensity histogram of the segmented area's pixels. The resulting image is *I*_4_ in Figure 3(a).

*(ii) Signal Identification and Tracking.* A detection and tracking algorithm has been developed to follow the motion of different signals as they traverse the FOV. Starting from the first appearance of a signal belonging to a track, defined as the time-series of occurrences of signals originating from the same particle, a search window centered on its centroid with dimensions of *S*_*ξ*_^*i*^|_*i*=1_ and *S*_*η*_^*i*^|_*i*=1_ is established. Here,

(2) $$\begin{array}{c} {\left.\;S_{\xi }^{i}\;\right|}_{i=1}={\left.\;S_{\eta }^{i}\;\right|}_{i=1}=k_{3}*\max\left\{\;B_{x}^{1},B_{y}^{1}\;\right\}, \end{array}$$

where *B*_*x*_^*i*^ and *B*_*y*_^*i*^ are the horizontal and vertical dimensions of the smallest rectangle bounding the enhanced particle image and *k*_3_ = 3 for the present datasets. The variables *ξ* and *η* are aligned in directions perpendicular and parallel to the signal motion, respectively, as shown in Figure 3(b). The next trace of the same particle is then searched within this window. Once the next trace is found, their intensity-weighted centroids are connected by a vector, *L*_1→2_. The size of subsequent windows is then modified to

(3) Sξi=k3∗Bji;Sηi=k3∗Li−1→i;i>1,

where *j* can be either *x* or *y*, depending on the coordinate most perpendicular to the direction of motion. The bottom edge of this window passes through the centroid of the latest trace and is aligned in the *ξ* direction.

A prescribed minimum number of matched traces along the same track are required for the signal to be considered valid. For the present relatively low-speed measurements, three traces are required, but different numbers might be more suitable for high-speed motions, for example. If the trajectory remains reasonably linear, the algorithm allows for detection and matching of traces in nonsequential exposures, that is, in the case of missing traces due to in- and out-of-plane motion. In the unlikely event of detecting more than one trace within a search window, the one with area closest to the average track area is selected. The resulting database is used for determining the number of distinct rVWF-eGFP multimers by accounting for each track once. Algorithms have also been developed for identifying the most in-focus occurrence in each track, fundamental for the evaluation of the rVWF-eGFP structural conformation. The selection is made based on the intensity gradients along the boundary of each detected trace in the original images *I*_0_, after median-filtering them with a window of 15 × 15 pixels to remove the salt-and-pepper noise while closely preserving the original intensity gradient. The median of the top twenty gradient values for each trace's boundary is used as a criterion for comparison and the highest one is selected.

#### 2.2.2. Activated Platelets

A postprocessing algorithm has been developed to quantify the number of activated platelets detected in deep samples. First, the images, which typically contain multiple traces, are median-filtered with a filter size of 15 × 15 pixels to remove the salt-and-pepper noise. Then, to increase the SNR, background nonuniformities are subtracted using running averages, as done for the rVWF-eGFP case. Since the SNR varies markedly among the traces, additional postprocessing is needed to distinguish between valid traces and noise. For the present cases, the platelets velocity in each image is nearly spatially uniform, presumably due to buoyant rise, providing additional information to distinguish between real and spurious signals. One velocity vector is calculated for each entire image using standard cross-correlation [34]. To remove spurious vectors, a smooth polynomial is fitted to the time history of velocity over the entire dataset. For the present data, the platelet velocity is in the range of 15–30 pixels/frame, corresponding to ~150–300 *μ*m/s. The velocity fluctuations around the fitted curve present a maximum of 5 pixels/frame and are due to the differences in motion of individual platelets compared to the bulk. To allow for such variation in the subsequent platelet matching, starting from the first image, the bounding boxes of each trace are padded with 5 pixels on each side and used as interrogation windows. The intensity distributions inside them are cross-correlated with corresponding areas in the previous and next two frames. The locations of correlation peaks with magnitude exceeding a threshold of 0.45 are compared to the expected displacement to determine whether matched traces exist in the neighboring frames. Sequences containing at least three consecutive traces are considered as confirmed signals. Then, the number of detected platelets per image is calculated for the entire dataset. In future studies involving nonuniform flows, one would not be able to rely on this procedure. As an alternative, each image could be exposed multiple times, and the reference velocity field could be determined by autocorrelation [34].

#### 2.2.3. VWF : IgG : FITC

The algorithms used for image acquisition and analysis of VWF-coated single platelets and VWF-platelet aggregates are the same as those employed for the rVWF-eGFP case. The same applies to the algorithms used for identifying the most in-focus trace in each track. In addition to this, the velocity of VWF-coated single platelets and VWF-platelet aggregates is evaluated. To do so, the area of the most in-focus image and those adjacent to it in time are calculated and compared. Only consecutive traces with an area change smaller than 25% of the median area value calculated around the most in-focus trace, ±2 images, are assumed to remain in focus and to be kept for velocity calculations. For the present samples, at least 5 consecutive traces need to satisfy this requirement. The velocity is determined based on the displacement of the centroid of the first and last exposure, thus reducing the inherent variability induced by the location of the centroid of each trace.

### 2.3. Sample Preparation

#### 2.3.1. Preparation of Washed Human Platelet Suspension

Whole blood from individually unidentifiable healthy human volunteers is collected in 1/10th volume of pH 5.3 citrate buffer and centrifuged at 145 g for 7 minutes at room temperature (21–25°C). Platelet-rich plasma (PRP) is then carefully collected and washed through one cycle of resuspension, using three volumes of Tyrode's buffer, pH 6.5, and one volume of citrate buffer, pH 5.3, and centrifugation at 640 g for 12 minutes at room temperature. At the low pH used during the isolation and washing procedure, platelet reactivity after stimulation is markedly decreased [35, 36]. Subsequently, the platelet pellet is resuspended in Tyrode's buffer, pH 7.4, after which 20 × 10^6^ washed platelets are incubated in a final volume of 200 *μ*l of Tyrode's buffer, pH 7.4, containing 0.2 *μ*g/ml anti-CD62P (anti-P-Selectin) antibody (Clone AK4, BioLegend) for 5 minutes at room temperature. The low proportion of washed platelets expressing P-Selectin without stimulation and the robust response after stimulation (see Figure 5) indicate that the washing procedure adequately protects platelet functional integrity. Unstimulated samples are prepared by diluting the platelet solution to a final concentration of 1000 platelet/*μ*l, suitable for imaging, using Tyrode's buffer, pH 7.4. For the stimulated samples, 20 × 10^6^ washed platelets are stimulated with thrombin (Haematologic Technologies, Inc.) with varying dosage in a final volume of 200 *μ*l of Tyrode's buffer, pH 7.4, containing 0.2 *μ*g/ml anti-CD62P antibody for 5 minutes at room temperature. This stimulated solution is also diluted to a concentration of 1000 platelet/*μ*l using Tyrode's buffer, pH 7.4. Image acquisition is performed immediately after sample preparation, followed by flow cytometry using an LSR II FACS machine. Both tests are completed within 30 minutes of the platelet activation procedure. In the case of Indo-1 labeling, platelets are incubated at room temperature for 30 minutes with 5 *μ*M Indo-1 in its cell-permeant AM form (Thermo Fisher Scientific, USA). The labeled platelets are then washed with Tyrode's buffer, pH 6.5, and centrifuged at 640 g for 12 minutes at room temperature. The platelet pellet is then labeled with the anti-CD62P antibody, and unstimulated and stimulated samples are prepared as previously described. Two experiments are performed. First, a repeatability test is performed. In this test, six platelet samples labeled with P-Selectin and one labeled with both P-Selectin and Indo-1 are evaluated with flow cytometry and the imaging system before and after stimulation with thrombin 5 nM. All samples are prepared from the same blood donor and at the same time. Second, platelets originating from a different donor are labeled with P-Selectin and stimulated with thrombin at concentrations increasing from 2.5 nM to 12.5 nM in steps of 2.5 nM. Three samples for each concentration are evaluated by the flow cytometer and the imaging system. Results of both experiments are incorporated for comparing the imaging and flow cytometry data. All studies have been approved by the Institutional Review Boards of the Johns Hopkins University and the Scripps Research Institute (study number IRB00044397).

#### 2.3.2. Synthesis and Analysis of rVWF-eGFP and Shear Tests

Human HEK-293 cells expressing rVWF-eGFP with HA- and His- tags [32] are cultured in OptiPRO™ with 1 mM sodium pyruvate, 2 mM L-glutamine, 100 U/mL penicillin, and 100 *μ*g/mL streptomycin. The media are collected, concentrated, and dialyzed. The concentration is performed using an Amicon Ultra-15 Centrifugal Filter Unit Ultra Cell-100 K (Millipore). The resulting solution is dialyzed using 10 K MWCO and 0.1–0.5 ml Slide-A-Lyzer cassettes (Thermo Fisher Scientific) spinning under magnetic stirring in 1 L of PBS at 4°C for 5 hours and then overnight after exchanging with fresh PBS. Aliquots are then prepared and stored at −80°C. On the day of the experiment, aliquots of rVWF-eGFP are thawed and diluted in PBS at the desired concentration. In the samples sheared in the presence of ADAMTS13, 2 *μ*g/ml of the enzyme (Abcam®) and 2 mM CaCl_2_ are added to the solution prior to the application of shear. EDTA is employed to inhibit ADAMTS13 activity. Brief bursts of shear are applied by forcing the solution through a 26Gx3/8′′ needle five times. Using the measured ejection time of 2 s and the total sample volume of 1.5 ml, the estimated maximum shear rate in the needle is about 10^5^ 1/s. Imaging is performed immediately after the final ejection. No modification to the binding activity of ADAMTS13 due to the attached eGFP molecule is expected, since the binding and cleavage site are spatially separated [37]. To observe isolated rVWF-eGFP filaments, the solution is first denatured using urea at a concentration of 1.5 M and then the buffer exchanged to recover the eGFP functionality. Imaging is then immediately performed. VWF antigen (VWF : Ag) is measured by enzyme-linked immunosorbent assay (ELISA) using anti-human VWF monoclonal antibody (2.2.9) as capture antibody and biotinylated anti-human VWF monoclonal antibody (5.5.72) as detection antibody. Purified recombinant human VWF protein was used to create a standard curve. Multimer analysis is performed to evaluate the presence/absence of proteolytic fragments. Details of the protocol are provided in Supplementary Material available online at https://doi.org/10.1155/2017/8318906.

#### 2.3.3. Human von Willebrand Factor Labeling with IgG : FITC and VWF-Platelet Solution

Cryoprecipitated human VWF and fresh PRP from unknown healthy human volunteers are obtained the day of the experiment from the Johns Hopkins Special Coagulation Laboratory. After dilution in PBS with 3% BSA, the VWF is incubated for 30 minutes with a sheep anti-human von Willebrand factor : FITC polyclonal antibody, IgG : FITC (AbD Serotec®), at a 1 : 10000 dilution. This dilution is chosen as it reduces the background fluorescence to a level allowing high SNR. PRP at 1 : 10 dilution and ristocetin, used to induce the initial VWF-platelet binding under static conditions [38], are then added to the labeled VWF solution. Thrombin is then introduced to promote platelet aggregation. Labeling the cryoprecipitated VWF instead of that present in the PRP enables tests using a well-controlled protein, without having to characterize the donor's VWF.

## 3. Results

### 3.1. Platelet Activation

**Figure 4:**
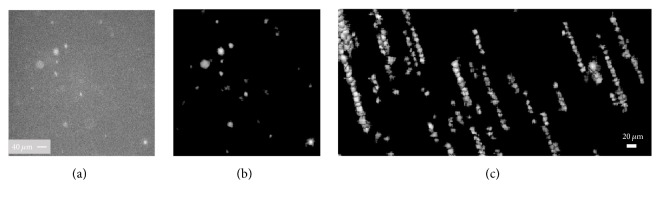
(a) Contrast adjusted raw image of activated platelets labeled with the anti-P-Selectin antibody and (b) the same image after postprocessing. Several signals, almost indistinguishable in the raw image, are recovered by the enhancement procedure. (c) Enhanced tracks of stimulated platelets visualized using Indo-1.

Figure 4(a) presents a characteristic contrast adjusted raw image of activated platelets labeled with the anti-P-Selectin antibody, and Figure 4(b) shows the same image after postprocessing. Of note, several traces which are barely observable in the original image are recovered and enhanced.  Figure 4(c) contains enhanced tracks, created by combining 30 consecutive frames, of stimulated Indo-1 loaded platelets. The spacing between consecutive signals is nearly constant and is equal for all the tracks, confirming the uniformity of the velocity field. This spatial uniformity justifies the use of the velocity field for distinguishing signal from noise, as previously described.

**Figure 5:**
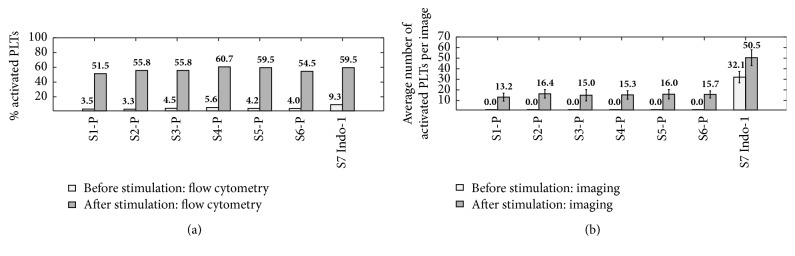
Comparison between (a) flow cytometry readings and (b) imaging data of six samples labeled with the anti-P-Selectin antibody (S1-P to S6-P) and one double-labeled platelet sample with Indo-1 and the anti-P-Selectin antibody (S7 Indo-1). Imaging data are represented as mean values with standard deviation bars superimposed. Stimulation is performed with thrombin 5 nM.

A repeatability test, aimed at confirming that consistent readings from the flow cytometer correspond to consistent results from the imaging system, is conducted using six P-Selectin labeled samples (S1-P to S6-P). In addition, to demonstrate the viability of using Indo-1 loaded platelets for detecting activation, one sample is double-labeled with the anti-P-Selectin antibody and with Indo-1. The fluorescence from the FITC-conjugated anti-P-Selectin antibody is employed in the flow cytometry measurements, while, in the imaging system, activation is detected by recording the light emitted at 405 nm, the peak wavelength for Ca^2+^-bound Indo-1 [39]. The results are presented in Figure 5. The average number and standard deviation of activated platelets per image are determined for all the 2000 images per dataset. Use of this average as a representative metric is justified by the presence of a single clear peak in the distribution of the number of activated platelets per image. The very low activation levels detected by flow cytometry before stimulation are reflected by the zero mean value detected by the imaging system. After activation, performed with thrombin 5 nM for all samples, an almost constant mean number of platelets are detected, consistent with the nearly constant activation level reported by the flow cytometer. The double-labeled sample (S7 Indo-1) also shows a clear increase in the number and percentage of activated platelets after stimulation. Notably, the difference between pre- and postactivation values for the double-labeled sample is consistent with that obtained with the P-Selectin ones. Hence, Indo-1 labeling could also be used as a method for detecting activation, particularly in light of its faster kinetics. The double-labeled case shows a nonzero image reading before activation. This is likely due to baseline [Ca^2+^]_i_ levels leading to the presence of Ca^2+^-bound Indo-1 inside platelets and/or to the presence of activated platelets showing increased [Ca^2+^]_i_ but still not expressing P-Selectin and, therefore, not detectable by the flow cytometer. The elevated baseline of the S7 Indo-1 sample in the flow cytometry results (9.3%) is likely caused by the additional manipulation required for the double-labeling.

**Figure 6:**
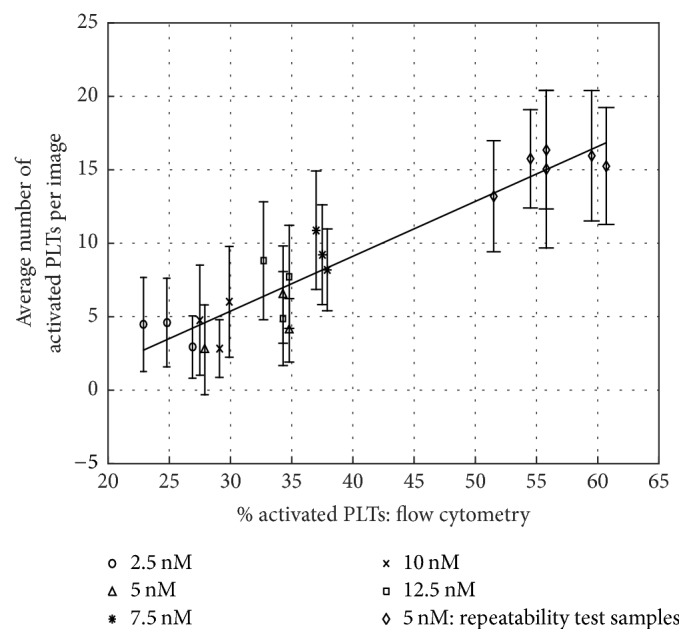
Correlation between the percentages of activated platelets detected with flow cytometry and the average number of activated platelets detected per image for all samples. Error bars show the standard deviations. The straight line represents the fit of the data.

To demonstrate the ability of the proposed techniques to detect varying levels of activated platelets consistent with flow cytometry readings, the samples are stimulated with different thrombin concentrations, as detailed in Methods. Results presented in Figure 6 incorporate all the data obtained in the two experiments with error bars representing the standard deviation of the imaging data for each set. They clearly show a positive correlation between the percentage of activated platelets detected with flow cytometry and the average number of activated platelets per image. In agreement with the consistency portrayed in Figure 5, the results obtained for each set of samples stimulated at the same thrombin concentration level show similar activation levels. However, it is important to underline that in the present data increasing the thrombin concentration does not result in a monotonic increase in platelet activation level. Various factors might contribute to this outcome, for example, P-Selectin shedding [40] and also the possibility that the stimulation with thrombin 2.5 nM is already excessive to explore the steepest part of the dose-response curve, which might lie between the 3.5% activation level before stimulation and the 23% of activation after stimulation with 2.5 nM. In this case, there would be no clear dose-response curve, thus explaining the scatter of the data. A detailed study of the observed behavior is beyond the scope of the present discussion. Yet, the clear agreement between flow cytometry and imaging results persists independently of the above-mentioned platelet activation trend. Lastly, note that the different response of the two donors' platelets to thrombin 5 nM is expected as the variability of the individual response to stimulation is a well-documented phenomenon [41].

**Figure 7:**
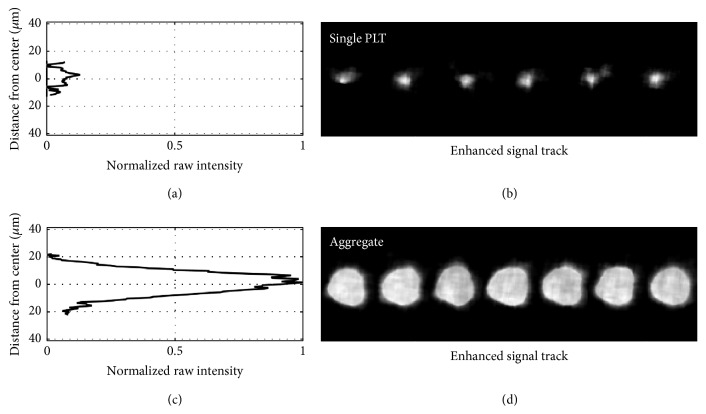
Normalized average raw intensity profiles ((a) and (c)) and enhanced signal tracks ((b) and (d)) of a single platelet ((a) and (b)) and a platelet aggregate ((c) and (d)). The platelet signal, significantly weaker than the aggregate one, is ~10 *μ*m in diameter, while the aggregate one is ~40 *μ*m. Both the single platelet and the aggregate are visualized using Indo-1.

The imaging technique can also readily detect platelet aggregates, a sample of which is shown in Figure 7 using platelets loaded with only Indo-1. These aggregates are much larger and brighter than single platelet traces, as indicated by the intensity profiles. These profiles are averaged over multiple traces and normalized using the peak value of the aggregate signal.

### 3.2. rVWF-eGFP

**Figure 8:**
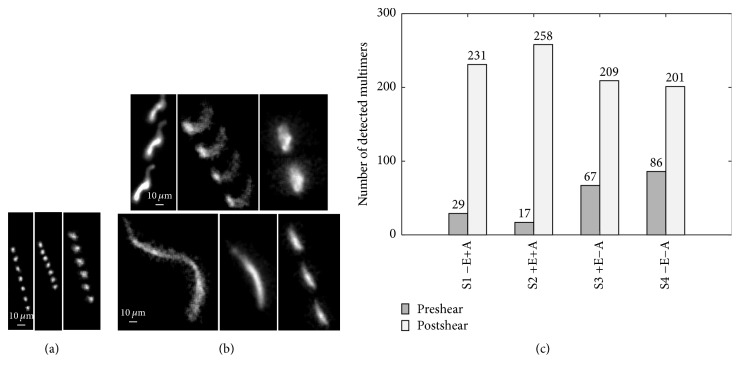
Enhanced signals and signal tracks of (a) globular rVWF-eGFP; (b) aggregates (top) and stretched filaments (bottom). Tracks are composed superimposing several occurrences of the same signal at a fixed time interval. Variation in the enhanced signal strength among different images is due to different levels of background noise and signal intensity in the raw images. (c) Number of distinct rVWF-eGFP multimers observed before (left) and after (right) application of shear in samples S1–S4. A substantial increase in the number of detected multimers after application of shear stress is observed in all cases. E: EDTA; A: ADAMTS13; ± indicates the presence/absence of the two compounds.

Figure 8(a) shows characteristic signals/tracks of rVWF-eGFP multimers in globular form, and Figure 8(b) displays aggregates and stretched filaments. The dimensions of the observed multimers in the various structural configurations are of the same order as those reported in literature [27, 29]. Formation of aggregates is consistent with previously reported evidence of VWF self-association under static conditions [42] and self-association of rVWF-eGFP in PBS [37]. For the presently analyzed cases, both rVWF-eGFP globules and aggregates are observed before and after application of shear; therefore the images of pre- and postshear samples appear to be qualitatively similar. Yet, the number of detected multimers is strikingly different, as discussed below. Stretched filaments are only observed under denaturing conditions. It should be emphasized that the samples are imaged only after, not during, the application of shear stress, possibly explaining the lack of stretched filaments in the postshear samples.

Four samples of rVWF-eGFP have been imaged before and after application of short bursts of shear stress to test the ability of the present techniques to detect changes in the number of rVWF-eGFP multimers as an indication of aggregation/disaggregation and/or cleavage. These tests have been performed in the presence/absence (±) of EDTA and/or ADAMTS13.  Figure 8(c) presents the number of distinct rVWF-eGFP multimers detected in 10000 images per dataset for these samples. The preshearing concentration for samples S1 and S2 is 3.0 *μ*g/ml, and for samples S3 and S4 it is 5.0 *μ*g/ml. The difference in concentration is a result of using two batches of rVWF-eGFP prepared at different times. After shearing, an expected loss of protein is observed. The concentration of samples S1 and S2 decreases to 2.0 *μ*g/ml, while that of S3 and S4 decreases to 4.0 *μ*g/ml. For all cases, application of shear markedly increases the number of detected multimers. The increase in sample S1, the only one which would allow mechanoenzymatic cleavage, is not significantly different from that of sample S2, where mechanoenzymatic cleavage is inhibited by the presence of EDTA. This trend suggests that this increase is not caused by cleavage. Indeed, no cleavage has been detected as indicated by the absence of proteolytic fragments in multimer analyses performed on the samples containing ADAMTS13 (see Supplementary Material). Furthermore, the increase in the number of signals occurs also in samples S3 and S4 that do not contain ADAMTS13. A likely explanation for these observations involves conformational changes associated with breakup of noncovalent bonds linking VWF aggregates but not covalent bonds within the multimers. The absence of cleavage even in the presence of ADAMTS13 suggests that the shear exposure time is much shorter than that required for exposing the A2 domain and for ADAMTS13 to cleave the protein. In other studies [37], exposure to the same level of shear for longer periods, more than 20 min versus the present few milliseconds for each passage through the needle, does cause substantial VWF cleavage. Nevertheless, the increase in the number of rVWF-eGFP multimers in each sample clearly appears to be a convenient way of detecting the breakup of aggregates resulting from short bursts of shear stresses. Such a short exposure to shear stress is encountered, for example, in stenotic cardiac valves and in left ventricular assist devices.

### 3.3. VWF : IgG : FITC

**Figure 9:**
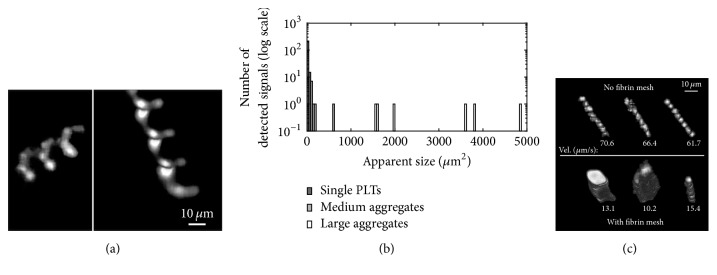
(a) Enhanced tracks of two VWF : IgG : FITC platelet-decorated aggregates. (b) Size distribution histogram presented in a log scale due to the high number of single platelets. (c) Enhanced tracks of single VWF-coated platelets without a fibrin mesh (top) and VWF-platelet aggregates within a fibrin mesh (bottom). The velocity in *μ*m/s is indicated under each track. In the latter case, the aggregates have a much lower velocity.

A PRP sample containing VWF : IgG : FITC is employed to detect VWF-platelet aggregates. To visualize isolated VWF-coated platelets, the sample is imaged first after adding ristocetin. Subsequently, thrombin is added, and the sample is imaged after one vortexing step lasting 10 s at 1500 RPM (Digital Vortex Genie 2, Scientific Industries). In both cases, 2000 images per dataset are recorded. The vortexing is aimed at providing enough mechanical excitation to promote, together with thrombin, platelet activation and aggregation. As shown in Figure 9(a), what seem to be VWF-platelet aggregates appear as bright regions connected by fainter ones, which likely represent VWF strands. The trace area distribution after excitation, presented in Figure 9(b), reveals three distinct sizes, representing single platelets (area < 85 *μ*m^2^), as well as medium (85 < area < 675 m^2^) and large aggregates (area > 675 *μ*m^2^). The size range representing isolated VWF-coated platelets is determined from the initial images recorded in the PRP sample with ristocetin, where no aggregates are present. To confirm the absence of aggregates due to platelet agglutination, the size of the platelets' signals from the PRP sample with ristocetin is compared with the ones from the platelets labeled with P-Selectin. Both samples show the same size distribution; therefore confirming the absence of platelet agglutination. As expected, the number of isolated VWF-coated platelets after excitation is still several orders of magnitude higher than those of the aggregates. Yet, the presence of medium- and large-scale aggregates is evident. In another sample prepared and stimulated the same way, the formation of what appeared to be a fibrin mesh is observed. This claim is made since the images become hazy, and the fibrin mesh is clearly visible by visual inspection. The VWF-platelet aggregates still form in this sample, but they are slowed down by the fibrin mesh compared to those in the mesh-free solution, even when they are of similar size.  Figure 9(c) compares characteristic tracks of VWF-coated platelets in fibrin-free solution to those of VWF-platelet aggregates within the fibrin mesh. As is evident, the velocity decreases on average by more than 80% in the latter case. These observations demonstrate the ability to characterize the effect of coagulation on the size and motion of suspended VWF-platelet aggregates.

## 4. Discussion

This paper introduces space and time resolved fluorescence imaging techniques for detecting in situ platelet activation, VWF structural conformational changes, and VWF-platelet aggregates moving in deep suspensions. These procedures expand currently available methodologies for observing coagulation-related processes and blood damage in static samples [32] and shallow channels [28, 29] and for wall-bound proteins and cells [25–27]. Detection and tracking of activated platelets are based on recording the fluorescence from a dye-conjugated anti-P-Selectin antibody and from Ca^2+^-bound Indo-1. A series of tests show that the average number of activated platelets per image positively correlates with flow cytometry measurements. The results are repeatable, proving the reliability of the new techniques. Recombinant VWF-eGFP multimers in different structural conformations are readily detected and tracked as they move in suspension. Detecting variations in the number of free-moving multimers in solution is an effective means of quantifying rVWF-eGFP conformational changes. Although the present measurements are restricted to breakup of aggregates due to the short shear exposure times, the same procedure could likely detect changes to the number of rVWF-eGFP multimers due to cleavage. The short exposures are meant to mimic scales relevant to passage through valves, left ventricular assist devices, and so forth. The use of VWF : IgG : FITC for detecting platelet-decorated VWF strands as they move in the solution allows for the quantification of changes to their shape and size, including the number of platelet aggregates. Consequently, this technique can visualize the effects of pro- and anticoagulant processes induced by shear stresses and/or chemical agonists/antagonists on the number and motion of aggregates. The latter is demonstrated here by showing the impact of fibrin mesh formation on the movement of VWF-platelet aggregates. These results are consistent with prior studies performed in microfluidic chambers and stenosis replica [8, 43, 44], where slowing down of platelets is used as an indicator of surface adhesion.

Detection of the phenomena discussed in this paper in deep suspensions introduces several challenges, primarily the low intensity of the fluorescent signals, which results in intensities that are just few gray levels above the noise floor. The presence of partially out-of-focus objects and the spatially and temporally varying background add further complexity. Consequently, data analysis requires ad hoc image enhancement and tracking algorithms aimed at increasing the low SNR. Edge-detection-based procedures are also needed for identifying the most in-focus signal within a track and for calculating geometric determinants, such as size, aspect ratio, and shape.

The present techniques can be applied in sample volumes with a half-depth falling within the working distance of the long-range microscope objectives employed.

**Figure 10:**
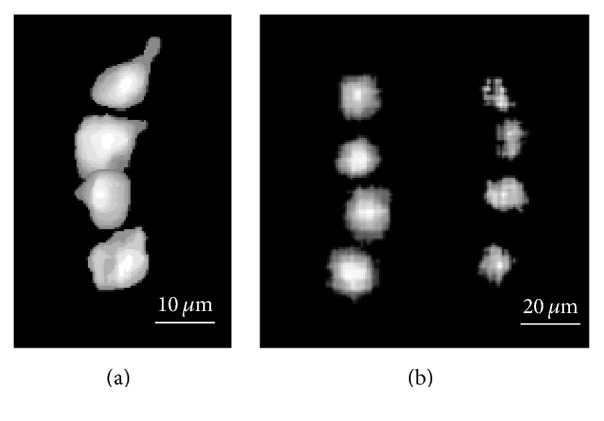
Enhanced tracks left by (a) an rVWF-eGFP multimer and (b) two activated Indo-1 loaded platelets recorded using a 1 ms exposure time.

Due to the high blood flow velocities encountered, for example, in stenosis, prosthetic heart valves, and left ventricular assist devices, the ability to record events using very short exposure times (≤1 ms) is fundamental. Application of high-sensitivity EM-CCD imaging enables us to achieve this goal, although most of the data presented in this paper involve longer exposure times. As a demonstration, Figure 10(a) shows sample track of an rVWF-eGFP multimer, and Figure 10(b) contains two sample tracks of activated platelets loaded only with Indo-1, all recorded using an exposure time of 1 ms. Further shortening of the exposure time might require more intense light sources than the present LEDs, for example, lasers. This would allow for a smaller illumination volume, therefore decreasing the background noise and hence facilitating measurement in more concentrated suspensions. The potential of photobleaching should be considered and evaluated. Its impact could be mitigated by controlling the illumination energy using proper selection of beam intensity, exposure time, and illuminated area. Moreover, the short transit times in high-speed flows of interest will also help in minimizing the effect of photobleaching. The number of frames per second of the camera can also be increased by reducing the image size, for example, 2053 fps for 64 × 64 pixels. Beyond this limit, multiple exposures could be recorded on the same frame, at least for low particle concentrations.

Finally, despite the fact that the presentation of the technique's capabilities has been focused on one cell and protein type, the introduced methods can be readily applied to other cell, protein, and flow types by appropriately choosing the postprocessing parameters in a way that best suits the particular application.

## 5. Conclusion

Platelet activation, VWF conformational changes, and VWF-platelet strands moving in deep suspensions are visualized and quantified using high-sensitivity fluorescent imaging. Several image enhancement and tracking procedures are applied to overcome low SNR conditions. The number of detected activated platelets is positively validated against standard flow cytometry measurements. The VWF conformational changes are readily quantified based on the number of multimers. Future tests will involve (i) systematic measurements of the effect of exposure time and shear level on the platelet and VWF response, (ii) comparison of the size and number of VWF-platelet aggregates to measurement obtained by standard aggregation tests [45], and (iii) application of the present techniques in transparent flow systems, such as heart valves and left ventricular assist device models.

## Supplementary Material

von-Willebrand Factor (VWF) multimer analysis of the four rVWF-eGFP imaged samples. NPP: normal pooled plasma.

## Figures and Tables

**Table 1:** Light sources (LEDs) and optical bandpass filters used for the different samples analyzed. ^*∗*^Peak wavelength. ^*∗∗*^Center wavelength/bandpass.

Sample	LED^*∗*^	Exc. filter^*∗∗*^	Em. filter^*∗∗*^
Platelet: Indo-1	340 nm	Not needed	400/25 nm
Platelet: P-Selectin	490 nm	482.5/31 nm	534.5/43 nm
rVWF-eGFP	470 nm	472/30 nm	525/50 nm
VWF : IgG : FITC	490 nm	482.5/31 nm	534.5/43 nm

## References

[1] Eckman P. M., John R. (2012). Bleeding and thrombosis in patients with continuous-flow ventricular assist devices. *Circulation*.

[2] Pareti F. I., Lattuada A., Bressi C. (2000). Proteolysis of von Willebrand factor and shear stress-induced platelet aggregation in patients with aortic valve stenosis. *Circulation*.

[3] Blitz A. (2014). Pump thrombosis-A riddle wrapped in a mystery inside an enigma. *Annals of Cardiothoracic Surgery*.

[4] Bartoli C. R., Restle D. J., Zhang D. M., Acker M. A., Atluri P. (2015). Pathologic von Willebrand factor degradation with a left ventricular assist device occurs via two distinct mechanisms: Mechanical demolition and enzymatic cleavage. *The Journal of Thoracic and Cardiovascular Surgery*.

[5] Birschmann I., Dittrich M., Eller T. (2014). Ambient hemolysis and activation of coagulation is different between HeartMate II and HeartWare left ventricular assist devices. *The Journal of Heart and Lung Transplantation*.

[6] Blann A. D., Nadar S. K., Lip G. Y. H. (2003). The adhesion molecule P-selectin and cardiovascular disease. *European Heart Journal*.

[7] Curvers J., De Wildt-Eggen J., Heeremans J., Scharenberg J., De Korte D., Van Der Meer P. F. (2008). Flow cytometric measurement of CD62P (P-selectin) expression on platelets: A multicenter optimization and standardization effort. *Transfusion*.

[8] Mazzucato M., Pradella P., Cozzi M. R., De Marco L., Ruggeri Z. M. (2002). Sequential cytoplasmic calcium signals in a 2-stage platelet activation process induced by the glycoprotein Ib*α* mechanoreceptor. *Blood*.

[9] Mazzucato M., Cozzi M. R., Pradella P., Ruggeri Z. M., De Marco L. (2004). Distinct roles of ADP receptors in von Willebrand factor-mediated platelet signaling and activation under high flow. *Blood*.

[10] Assinger A., Volf I., Schmid D. (2015). A Novel,rapid method to quantify intraplatelet calcium dynamics by ratiometric flow cytometry. *PLoS ONE*.

[11] Oliver A. E., Tablin F., Walker N. J., Crowe J. H. (1999). The internal calcium concentration of human platelets increases during chilling. *Biochimica et Biophysica Acta (BBA) - Biomembranes*.

[12] Chow T. W., Hellums J. D., Moake J. L., Kroll M. H. (1992). Shear stress-induced von willebrand factor binding to platelet glycoprotein Ib initiates calcium influx associated with aggregation. *Blood*.

[13] Rink T. J., Sage S. O. (1990). Calcium signaling in human platelets. *Annual Review of Physiology*.

[14] Siffert W., Siffert G., Scheid P., Akkerman J. W. N. (1989). Activation of Na+/H+ exchange and Ca2+ mobilization start simultaneously in thrombin-stimulated platelets. Evidence that platelet shape change disturbs early rises of BCECF fluorescence which causes an underestimation of actual cytosolic alkalinization. *Biochemical Journal*.

[15] Grynkiewicz G., Poenie M., Tsien R. Y. (1985). A new generation of Ca^2+^ indicators with greatly improved fluorescence properties. *The Journal of Biological Chemistry*.

[16] Huck V., Schneider M. F., Gorzelanny C., Schneider S. W. (2014). The various states of von Willebrand factor and their function in physiology and pathophysiology. *Thrombosis and Haemostasis*.

[17] Mendolicchio G. L., Ruggeri Z. M. (2005). New perspectives on von Willebrand factor functions in hemostasis and thrombosis. *Seminars in Hematology*.

[18] Ruggeri Z. M. (2007). The role of von Willebrand factor in thrombus formation. *Thrombosis Research*.

[19] Sadler J. E. (1998). Biochemistry and genetics of von Willebrand factor. *Annual Review of Biochemistry*.

[20] Dong J.-F., Moake J. L., Nolasco L. (2002). ADAMTS-13 rapidly cleaves newly secreted ultralarge von Willebrand factor multimers on the endothelial surface under flowing conditions. *Blood*.

[21] Nesbitt W. S., Westein E., Tovar-Lopez F. J. (2009). A shear gradient-dependent platelet aggregation mechanism drives thrombus formation. *Nature Medicine*.

[22] Chauhan A. K., Goerge T., Schneider S. W., Wagner D. D. (2007). Formation of platelet strings and microthrombi in the presence of ADAMTS-13 inhibitor does not require P-selectin or *β*3 integrin. *Journal of Thrombosis and Haemostasis*.

[23] Costello J. P., Diab Y. A., Philippe-Auguste M. (2014). Acquired von Willebrand Syndrome in a Child Following Berlin Heart EXCOR Pediatric Ventricular Assist Device Implantation: Case Report and Concise Literature Review. *World Journal for Pediatric and Congenital Heart Surgery*.

[24] Crow S., Chen D., Milano C. (2010). Acquired von Willebrand syndrome in continuous-flow ventricular assist device recipients. *The Annals of Thoracic Surgery*.

[25] Colace T. V., Diamond S. L. (2013). Direct observation of von Willebrand factor elongation and fiber formation on collagen during acute whole blood exposure to pathological flow. *Arteriosclerosis, Thrombosis, and Vascular Biology*.

[26] De Ceunynck K., Rocha S., Feys H. B. (2011). Local Elongation of Endothelial Cell-anchored von Willebrand Factor Strings Precedes ADAMTS13 Protein-mediated Proteolysis. *The Journal of Biological Chemistry*.

[27] Zheng Y., Chen J., López J. A. (2015). Flow-driven assembly of VWF fibres and webs in in vitro microvessels. *Nature Communications*.

[28] Vergauwe R. M. A., Uji-I H., De Ceunynck K., Vermant J., Vanhoorelbeke K., Hofkens J. (2014). Shear-stress-induced conformational changes of von Willebrand factor in a water-glycerol mixture observed with single molecule microscopy. *The Journal of Physical Chemistry B*.

[29] Schneider S. W., Nuschele S., Wixforth A. (2007). Shear-induced unfolding triggers adhesion of von Willebrand factor fibers. *Proceedings of the National Acadamy of Sciences of the United States of America*.

[30] Singh I., Themistou E., Porcar L., Neelamegham S. (2009). Fluid shear induces conformation change in human blood protein von Willebrand factor in solution. *Biophysical Journal*.

[31] Shankaran H., Alexandridis P., Neelamegham S. (2003). Aspects of hydrodynamic shear regulating shear-induced platelet activation and self-association of von Willebrand factor in suspension. *Blood*.

[32] Lippok S., Obser T., Müller J. P. (2013). Exponential size distribution of von Willebrand factor. *Biophysical Journal*.

[33] Lim J. S. (1988). Two-dimensional signal processing. *Advanced topics in signed processing*.

[34] Estruch-Samper D. (2012). Particle Image Velocimetry, R. J. Adrian and J. Westerweel, Cambridge University Press, The Edinburgh Building, Shaftesbury Road, Cambridge, CB2 2RU, UK. 2011. 558pp. £75. ISBN 978-0-521-44008-0.. *The Aeronautical Journal*.

[35] Scharbert G., Franta G., Wetzel L., Kozek-Langenecker S. (2011). Effect of pH levels on platelet aggregation and coagulation: a whole blood in vitro study. *Critical Care*.

[36] Green F. W., Kaplan M. M., Curtis L. E., Levine P. H. (1978). Effect of acid and pepsin on blood coagulation and platelet aggregation. A possible contributor prolonged gastroduodenal mucosal hemorrhage. *Gastroenterology*.

[37] Lippok S., Radtke M., Obser T. (2016). Shear-Induced Unfolding and Enzymatic Cleavage of Full-Length VWF Multimers. *Biophysical Journal*.

[38] Coller B. S., Gralnick H. R. (1977). Studies on the mechanism of ristocetin induced platelet agglutination. Effects of structural modification of ristocetin and vancomycin. *The Journal of Clinical Investigation*.

[39] Takahashi A., Camacho P., Lechleiter J. D., Herman B. (1999). Measurement of intracellular calcium. *Physiological Reviews*.

[40] Marder V. J., Aird W. C., Bennett J. S. (2013). *Basic Principles and Clinical Practice*.

[41] Geisler T., Schaeffeler E., Gawaz M., Schwab M. (2013). Genetic variation of platelet function and pharmacology: An update of current knowledge. *Thrombosis and Haemostasis*.

[42] Ulrichts H., Vanhoorelbeke K., Girma J. P., Lenting P. J., Vauterin S., Deckmyn H. (2005). The von Willebrand factor self-association is modulated by a multiple domain interaction. *Journal of Thrombosis and Haemostasis*.

[43] Jamiolkowsi M. Real-time analysis of flow-induced thrombus formation within a defined crevice.

[44] Para A. N., Ku D. N. (2013). A low-volume, single pass in-vitro system of high shear thrombosis in a stenosis. *Thrombosis Research*.

[45] Gibbins J. M., Mahaut-Smith M. P. (2004). *Platelets and Megakaryocytes*.

