# Supplementary material for: Space and Time Resolved Detection of Platelet Activation and von Willebrand Factor Conformational Changes in Deep Suspensions
Source: Int J Biomed Imaging. 2017 Nov 6;2017:8318906. doi: 10.1155/2017/8318906 (PMC5695078; doi:10.1155/2017/8318906)
Supplement: Supplementary file 1 — von-Willebrand Factor (VWF) multimer analysis of the four rVWF-eGFP imaged samples. NPP: normal pooled plasma. [file 8318906.f1.pdf]

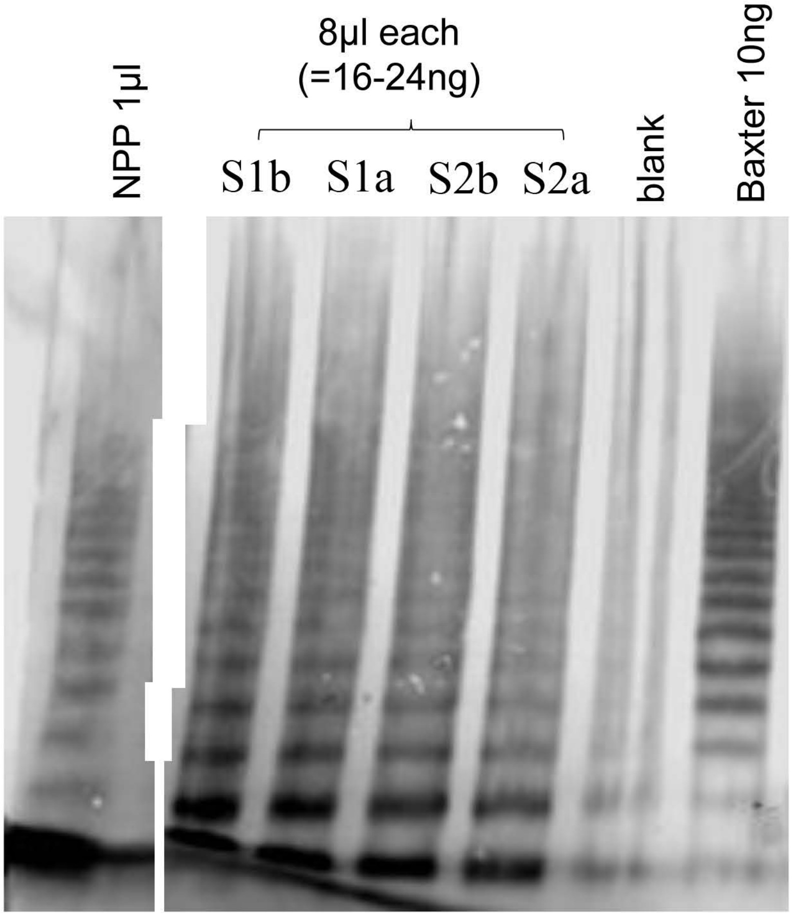

Biorad gel tray; 1.0% HGT(P); 30ml

Electrophoresis 40mA for 3 hours
Transfer at 400mA for 4 hours

1st Ab; anti-VWF pAb 1: 2,000 for O/N
2nd Ab: LiCOR anti-rabbit 1:20,000 for 1 hour

S1b **no EDTA no ADAMTS13, before shear; 3µg/ml**
S1a **no EDTA with ADAMTS13, after shear; 2µg/ml**
S2b **+ EDTA no ADAMTS13, before shear; 3µg/ml**
S2a **+ EDTA with ADAMTS13, after shear; 2µg/ml**

Western blotting

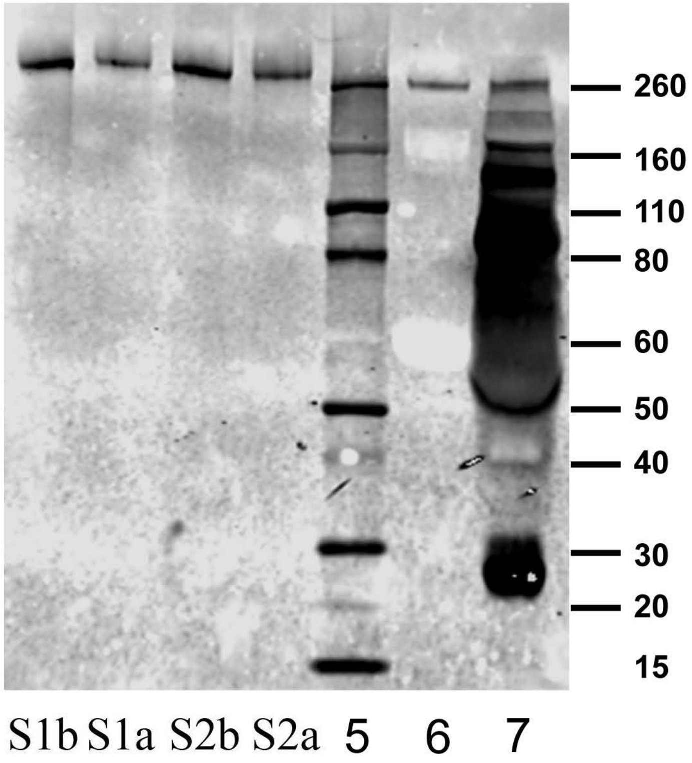

4-12% Tris-Bis Reduced
anti-VWF pAb

S1b **no EDTA no ADAMTS13, before shear; 3µg/ml**
S1a **no EDTA with ADAMTS13, after shear; 2µg/ml**
S2b **+ EDTA no ADAMTS13, before shear; 3µg/ml**
S2a **+ EDTA with ADAMTS13, after shear; 2µg/ml**

loaded 20 µl each (=40-60ng)

5- MW Marker

6- rVWF from Baxter (20ng)

7- NPP (2 µL)
